# Diagnostic and Predictive Values of LAP in Hypertension: A Cross-Sectional Study in Chinese Population Older Than 65 Years

**DOI:** 10.1155/2021/3066007

**Published:** 2021-09-21

**Authors:** Shuo Yan, Qing-Hu Zheng, Dong-Mei Sun, Ying Wu, Tu-Ming Li, Ping Zhong

**Affiliations:** ^1^Shanghai Medical College of Integrated Traditional Chinese and Western Medicine, Shanghai University of Traditional Chinese Medicine, Shanghai, China; ^2^Department of Neurology, Shidong Hospital, Yangpu District, Shanghai, China; ^3^Shanghai University of Traditional Chinese Medicine, Shanghai, China; ^4^Puxing Community Health Service Center, Pudong New Area, Shanghai, China

## Abstract

This study aimed to investigate the predictive value of lipid accumulation product (LAP) in hypertension in Chinese population older than 65 years. A total of 2092 adults from the communities in Pudong New Area of Shanghai were included in this cross-sectional study. The participants filled in questionnaire and received anthropometric and laboratory examinations. The receiver operating characteristics curve (ROC) was used to analyze the predictive value of different risk factors in hypertension. Results showed that LAP was closely related to hypertension (adjusted OR: 1.011, 95% CI: 1.007–1.015). In females, LAP, fasting blood glucose (FPG), and body mass index (BMI) were associated with hypertension; in males, triglycerides (TG) and waist circumference (WC) were related to hypertension. LAP (AUC = 0.655, 95% CI: 0.632–0.679) was better than neck circumference (NC) and BMI in predicting hypertension. When the cutoff value was 33.5, LAP had the best predictive performance. In males, LAP at 36.72 and 56.76 had the best predictive performance in males (AUC = 0.663, 95% CI: 0.629–0.697) and females (AUC = 0.650, 95% CI: 0.618–0.682), respectively. In conclusion, LAP is a risk factor of hypertension in the elderly. For hypertension, BMI, FPG, and LAP have favorable predictive performance in females, and WC and TG have better predictive performance in males.

## 1. Introduction

Hypertension, a significant risk factor for cardiovascular diseases and renal disorders, has been one of the most prevalent global public health problems and imposes considerable burden to the global health [1].

Obesity, abnormal accumulation lipid has been confirmed to be harmful for health, and it can raise angiotensin and aldosterone in the chronic hypoxemia population through activating fat cells, which then increases the risk for hypertension [2, 3]. It has been reported that abdominal or visceral fat is superior to subcutaneous fat in the prediction of hypertension [4]. However, body mass index (BMI) is a limited indicator for the identification of anatomical location or function of different fat deposits. Kahn proposed the concept of lipid accumulation product (LAP) at the beginning of twenty-first century, and the lipid accumulation index has been used as a marker of the degree of abdominal obesity based on the ratio of waist circumference (WC) and triglyceride (TG) content [5], and it was calculated as (WC−60.6) × (TG [mmol/L]) in males and (WC−54.1) × (TG [mmol/L]) in females based on the findings from Shanghai Chinese population. In addition, LAP has been found to be also closely related to type 2 diabetes [6], cardiovascular diseases [7], hypertension [8], and ovarian syndrome [9]. LAP may be better to predict the risk of a disease according to the fat content and distribution. This study aimed to investigate the predictive value of LAP in hypertension in Chinese population older than 65 years.

## 2. Materials and Methods

### 2.1. Subjects

This was a cross-sectional study with sampling survey and subjects were recruited from 11 communities in the Pudong New Area of Shanghai between January 2012 and March 2012. A total of 2092 subjects filled in the questionnaire. The mean age was 73.21 ± 6.71 years, and 46.7% was males. The inclusion criteria were as follows: subjects who lived in the communities for more than 5 years and subjects aged over 65 years and volunteered to participate in this study. The exclusion criteria were as follows: subjects who had thyromegaly or severe systemic diseases (such as liver and kidney dysfunction, cancer, heart failure, or acquired immune deficiency syndrome) and subjects who were bodybuilder, or professional or amateur athletes. All participants signed informed consent before the study.

### 2.2. Collection of Clinical Information

The following clinical information was collected via a standard questionnaire: age, gender, smoking status, alcohol consumption, time of physical exercise, prior hypertension, and history of diabetes and coronary heart disease (CHD). A current smoker was defined as a person who reported smoking every day or every few days and smoked at least 100 cigarettes in lifetime. Alcohol use was defined as that a person drank alcohol containing liquid more than 20–30 g (man) or 10–20 g (woman) every day. Hypertension was defined as systolic blood pressure (SBP) ≥ 140 mmHg or diastolic blood pressure (DBP) ≥ 90 mmHg or a history of oral antihypertensive medication. CHD was defined as coronary artery stenosis of up to 50% on coronary angiography or a history of acute myocardial infarction. Diabetes was defined as fasting plasma glucose (FPG) ≥ 6.1 mmol. WC was measured through the horizontal circumference at the center of the umbilicus. Neck circumference (NC) was measured as the horizontal circumference of the neck below the Adam's apple. Height and weight were measured, and the weight-height ratio (WHR), weight-neck ratio (WNR), and BMI were calculated. Total cholesterol (TC), TG, and total bilirubin (TBIL) were used as usual indicators of lipid metabolism and reference ranges were as follows: TC: 3.1–5.2 mmol/L, TG: 0.45–1.69 mmol/L, TBIL: 1.7–17.1 *μ*mol/L.

### 2.3. Anthropometry

The subjects were in light clothes and without shoes when the height and weight were measured. They were measured 2 times with an interval of 10 minutes and the average was calculated during the measurement of blood pressure. The WC was measured at the level between the lower rib margin and the crista iliac. When the NC was measured, the head stretched straight, the eyes looked forward, and the horizontal measurement was conducted at the upper margin of the laryngeal prominence. BMI was calculated as weight (kg) divided by the square of height (m).

### 2.4. Blood Biochemical Detections

Fasting venous blood was obtained and processed for the measurement of FPG, TG, TC, and TBIL. Blood samples were stored at −80°C before detections.

### 2.5. Statistical Analysis

Quantitative data are expressed as mean ± standard deviation (SD) or median/interquartile range and qualitative data as ratio. The Kolmogorov–Smimov test was applied to assess the normality of the data. The differences of quantitative data among patients with different hypertension statuses were analyzed with the Kruskal–Wallis H test when the data had no normal distribution. Categorical variables were analyzed with the Chi square test. Logistic stepwise regression was used to explore the risk factors with statistical significance for hypertension, and binary logistic regression to analyze the influence of each risk factor on hypertension. The association of LAP with other risk factors of hypertension was subsequently analyzed using the Spearmen correlation. The receiver operating characteristic (ROC) curve was used to analyze the predictive value of each risk factor on hypertension, and a value of *P* < 0.05 was considered statistically significant. Statistical analyses were performed using the Statistical Product and Service Solutions (SPSS) version 26.0 (SPSS Inc., Chicago, IL, USA).

## 3. Results

### 3.1. Characteristics of Subjects at Baseline

A total of 2092 adults were enrolled into this study. The average age was 73.21 ± 6.711 years. There were 971 males and 1121 females. Among them, 1026 subjects had a history of hypertension, and 1066 had no history of hypertension. There was no significant difference in hypertension between men and women. Significant differences were noted in the age (*P* < 0.001), history of CHD (*P* < 0.001), and diabetes (*P* < 0.001) between subjects with hypertension and those without hypertension. There were no marked differences in the smoking status and drinking status between subjects with and without hypertension. In addition, there were significant differences in the body weight (*P* < 0.001), WC (*P* < 0.001), NC (*P* < 0.001), BMI (*P* < 0.001), WHR (*P* < 0.001), and WNR (*P* < 0.001) between subjects with and without hypertension. Marked differences were also observed in body weight, WC, NC, WHR, and WNR between males and females. Laboratory examinations showed FGP (*P* < 0.001), TG (*P* < 0.001), alanine aminotransferase (ALT) (*P* < 0.001), and LAP (*P* < 0.001) were markedly different between subjects with and without hypertension. There were significant differences in the TG (*P* < 0.001), TBIL (*P* < 0.001), TC (*P* < 0.001), and LAP (*P* < 0.001) between males and females (Tables 1 and 2).

### 3.2. Risk Factors of Hypertension

**Figure 1:**
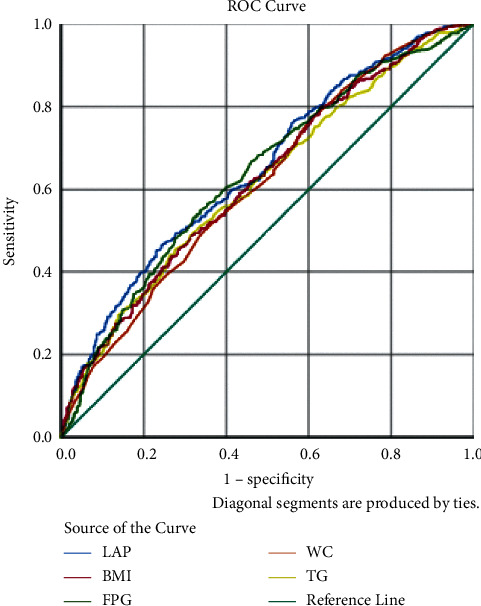
ROC curve in female.

Binary logistic regression was also used to screen related factors. Results showed that, in all the subjects, LAP (*P* < 0.001), FPG (*P* < 0.01), BMI (*P* < 0.01), and NC (*P* < 0.001) were associated with hypertension. In addition, LAP (*P* < 0.001), FPG (*P* < 0.01), and BMI (*P* < 0.001) were closely related to hypertension in females. However, LAP was not associated with hypertension in males. WC (*P* < 0.001) and TG (*P* < 0.001) were closely related to hypertension in males (Figure 1).

In the multivariate logistic regression analysis, after adjustment for gender, age, and other confounding factors, LAP had a close relationship with the history of hypertension (adjusted OR: 1.011, 95% CI: 1.007–1.015, *P* < 0.001), but the risk was relatively low, and there was marked difference between males and females (male, OR: 1.011, 95% CI: 1.005–1.017, *P* < 0.01; female, OR: 1.012, 95% CI: 1.007–1.017, *P* < 0.001). In addition, in males, TG (*P* < 0.001) and WC (*P* < 0.001) had a close relationship with hypertension as compared to LAP (*P* < 0.01). However, in females, FPG (*P* < 0.01), LAP (*P* < 0.001), BMI (*P* < 0.001), WC (*P* < 0.001), and TG (*P* < 0.001) were closely related to hypertension (Tables 3–5).

**Figure 2:**
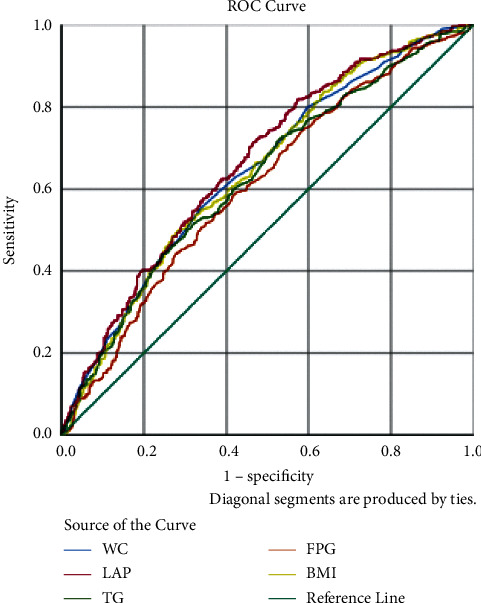
ROC curve in male.

**Figure 3:**
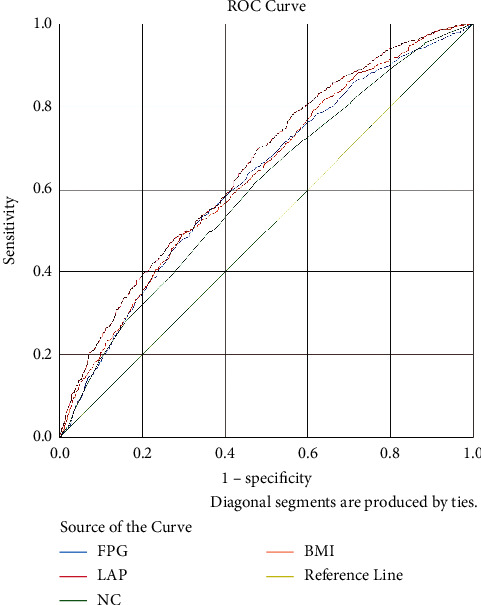
ROC curve of overall.

The ROC curves are shown in Figures 1–3 and Table 6. The predictive performance of LAP in hypertension was better than the remaining risk factors in males, females, and all the subjects. In all the subjects, the AUC of LAP was 0.655 (95% CI: 0.632–0.679), which was higher than those of NC (AUC = 0.601, 95% CI: 0.576–0.625), FPG (AUC = 0.624, 95% CI: 0.600–0.648), and BMI (AUC = 0.632, 95% CI: 0.608–0.655). LAP at 33.5 had the best predictive performance. In males, the AUC of LAP was 0.663 (95% CI: 0.629–0.697), which was higher than those of other four factors. The predictive performance of WC (AUC = 0.644, 95% CI: 0.609–0.678) was better than that of TG (AUC = 0.626 95% CI: 0.591–0.661); the AUC of BMI (0.642, 95CI% 0.608–0.677) was slightly lower than that of WC, but higher than that of FPG (AUC = 0.608, 95% CI: 0.573–0.643). As shown in Table 7, results showed that LAP *vs.* NC (*χ*^2^  = 16.2, *P* < 0.001), LAP *vs.* FPG (*χ*^2^ = 5.09, *P* < 0.05), and LAP *vs.* BMI (*χ*^2^  = 5.10, *P* < 0.01) were significant separately. LAP at 36.72 had the best predictive performance. In females, the order of predictive performance was LAP (AUC = 0.650, 95% CI: 0.618–0.682), FPG (AUC = 0.637, 95% CI: 0.605–0.670), BMI (AUC = 0.624, 95% CI: 0.592–0.657), WC (AUC = 0.616, 95% CI: 0.584–0.649), and TG (AUC = 0.617, 95% CI: 0.585–0.650), and LAP at 56.76 had the best predictive performance.

The comparison of each indicator in ROC analysis showed LAP (AUC = 0.655) *vs.* BMI (AUC = 0.632). FPG (AUC = 0.624) and NC (AUC = 0.601) had significant differences (Table 7). The incremental performance analysis in Table 8 showed that the *R*^2^ of LAP (Cox and Shell *R*^2^ = 0.65, Nagelkerke *R*^2^ = 0.86) alone was inferior to the combinations of LAP + BMI (Cox and Shell *R*^2^ = 0.77, Nagelkerke *R*^2^ = 0.102), LAP + NC (Cox and Shell *R*^2^ = 0.73, Nagelkerke *R*^2^ = 0.98), LAP + TG (Cox and Shell *R*^2^ = 0.67, Nagelkerke *R*^2^ = 0.89), LAP + WC (Cox and Shell *R*^2^ = 0.74, Nagelkerke *R*^2^ = 0.99), and LAP + FPG (Cox and Shell *R*^2^ = 0.71, Nagelkerke *R*^2^ = 0.95). Logistic regression analysis indicated that there was significant correlation (*P* < 0.001) between history of hypertension and LAP (OR = 2.533, 95% CI: 2.117–3.031) which was converted into two categories according to the cutoff value (33.5).

## 4. Discussion

With the economic development and the change in lifestyle, hypertension has gradually become a serious public health problem [10]. The pathology can involve multiple organs in patients with hypertension, and the common complications of hypertension include renal failure, stroke, and cardiomyopathy caused by cardiovascular factors [11]. In China, the increase in the incidence of hypertension in rural areas is much higher than in urban areas [12]. There is evidence showing that visceral fat can cause high blood pressure through inducing sodium retention, insulin resistance, renin-angiotensin-aldosterone activation, alteration of vascular function, and secretion of related adipokines [13].

Subcutaneous fat and visceral fat are adipose tissues that play distinct roles in the metabolism. Subcutaneous fat is a protective and safe fat storage, while visceral fat is harmful [14]. Prospective studies have shown that the abdominal obesity is an independent metabolic disorder and a risk factor of mortality as compared to the BMI and whole-body fat [15]. In subjects with abdominal obesity, visceral adipose tissue (VAT) plays an important role in the pathogenesis of metabolic diseases and cardiovascular diseases [14]. Even in nonobese people, the accumulation of VAT may still exert harmful effect. Studies have shown that the traditional obesity-related factors such as BMI and WC have limitations in the assessment of visceral or abdominal fat [8]. LAP is based on the WC and TG and can well reflect the accumulation of visceral and abdominal fat. Kahn for the first time proposed the concept of LAP in 2015 and found that it was superior to BMI in identifying cardiovascular risks. Thereafter, increasing studies have been conducted to investigate the predictive values of LAP in diseases [5]. In most studies, LAP is calculated in the European and American, and there are significant individual differences between Chinese and European and American. In the present study, LAP was calculated according to the formula based on the accumulation of visceral and abdominal fat tissues in the Chinese provided by Huang et al. In recent years, LAP was been found to be related to the pathogenesis of cardiovascular diseases, and it also serves as a good predictor of hypertension [16].

In the study of Huang et al., LAP had diagnostic and predictive values for hypertension. In their study, the average age was 41 years, and 23.7% was the elderly. In the present study, the OR value showed the relationship between LAP and hypertension, and LAP had good predictive performance on hypertension, but the risk was relatively small as compared to it. This may be explained as the difference in the included populations among studies. In the present study, subjects older than 65 years were recruited from Shanghai. In the elderly, the elasticity of large arteries is reduced, the ability of the kidneys to maintain ion balance is also significantly compromised, and the reflex sensitivity of baroreceptor is reduced, all of which lead to the increases in the peripheral resistance and the risk of hypertension in the elderly [17]. In 1991, a Chinese epidemiological survey showed that the prevalence of hypertension was 40.4% in the subjects older than 60, and it increased to 53.2% in 2015. Kahn et al. found that LAP was decreasing over years in males, accompanied by the increase in the prevalence of hypertension. This may be one of the reasons that LAP was not associated with LAP in the male subjects older than 65 years. This also suggests that the formula for LAP should be optimized in the elderly.

The ROC showed that the LAP, BMI, and FPG had good predictive performance on the risk of hypertension in males, but FPG had no close relationship with hypertension after adjustment. However, in the univariate logistic analysis, the LAP, BMI, and FPG were closely related to hypertension in males. This indicates that this indicator has diagnostic and predictive values on hypertension. Analysis of interaction between FPG and LAP, WHR, WNR, BMI, NC, and TG by turn showed FPG was affected mainly by some factors such as NC, TG, BMI, and LAP, but the significant difference was absent after adjustment. A large number of studies have shown that LAP has a favorable predictive performance on hypertension [16, 18]. This suggests that although LAP has diagnostic value on hypertension, it may seem reluctant as an independent predictor of hypertension in the present study. In addition, the predictive and diagnostic performance on hypertension was found to be different between males and females. That is, BMI, FPG, and LAP we significantly different in women, while WC and TG were comparable in females. The opposite results were found in males: significant differences were observed in the WC and TG, while marked differences were not observed in the BMI, FPG, and LAP after adjustment.

In a study which investigated the correlation between obesity and atrial fibrillation, results showed that BMI could more stably and comprehensively predict the risk of atrial fibrillation in women, but WC served as a better predictor in men [19]. Hypertension and obesity have also been proven as risk factors of atrial fibrillation [20]. These findings may provide evidence on the differences between males and females in the present study: the predictive factors related to hypertension or cardiovascular diseases are different between males and females. In a study on the correlation between diabetes and obesity, central obesity based on WC and WHR was found as a more specific risk factor of diabetes in males as compared to that in females [21, 22]. In the present study, the FPG and BMI were more closely related to hypertension in females than in males.

A cross-sectional survey showed that the FPG in patients without diabetes was significantly different between males and females [23]. The lifelong social and psychological factors (such as gender-sensitive economic, behavioral, cultural, and environmental factors) may also contribute to the differences between males and females besides the differences in the sex hormones and the expression of gender-specific genes [24, 25]. Increasing studies have shown that FPG can be used to predict hypertension [26, 27]. Our study also showed that FPG was related to hypertension and had predictive value on hypertension in the Chinese elderly. As compared with the relationship between FPG and hypertension in females, FPG had no relationship with hypertension in males after adjustment.

NC is the external reflection of neck adipose tissue (NAT) and reflects the subcutaneous fat deposit in the upper body. Although NAT belongs to the subcutaneous fat, it can serve as an independent disease-causing fat deposit or may explain the additional risk that cannot be predicted by the VAT [28]. When it is used to predict the risk of obesity and fat-related diseases, it can avoid the interference of breathing, food intake, and clothes as compared to WC, and it has been considered to be a reliable indicator of upper body SAT [29, 30]. In a study involving 5209 subjects aged 28–62 years, results showed NC was closely related to hypertension, diabetes, and dyslipidemia, and this correlation was more evident in the females [31]. In addition, NC is also related to a variety of cardiovascular risk factors and cardiovascular diseases [32, 33]. In the present study, the correlation between NC and hypertension was observed in the Chinese elderly, and NC had predictive value on hypertension. However, the predictive value of NC was not observed in males or females alone.

The present study still had several limitations. First, the sample size was small, and the causal relationship between each predictor and hypertension was not further investigated. In addition, only subjects from Shanghai were included into present study, and more studies are needed to investigate the predictive value of LAP on hypertension in other areas of China.

## 5. Conclusions

LAP is closely associated with the risk of hypertension in the elderly. In the prediction of the risk of hypertension, the performance of BMI, FPG, and LAP is better in women, but the performance of WC and TG is better in men.

## Figures and Tables

**Table 1:** Baseline characteristics of subjects.

Variables	Normotension (*n* = 1026)	Hypertension (*n* = 1066)	*χ*^2^/*Z*	*P*
Sex (%)			1.777	0.182
Male	23.5%	23.2%		
Female	25.2%	28%		
Age (years)	72.94 ± 6.897	73.45 ± 6.520	−2.499	0.012
Smoker (%)	7.4	6.6	2.764	0.096
Drinker (%)	8.3	7.6	2.123	0.145
History of CHD	5.3	11	42.544	<0.001
History of diabetes	4.9	11.7	60.938	<0.001
Height (cm)	160.08 ± 8.431	160.31 ± 8.406	−0.308	0.758
Body weight (kg)	59.177 ± 10.641	63.351 ± 10.742	−8.752	<0.001
WC	84.699 ± 10.071	88.789 ± 9.152	−9.504	<0.001
NC	35.46 ± 3.241	36.525 ± 3.191	−7.549	<0.001
BMI	22.956 ± 3.514	24.587 ± 3.428	−10.418	<0.001
WHR	0.53 ± 0.066	0.55 ± 0.592	−9.516	<0.001
WNR	2.39 ± 0.048	2.44 ± 0.037	−5.577	<0.001
SBP	129.5 ± 14.766	139.63 ± 15.408	−14.588	<0.001
DBP	76.19 ± 8.033	79.57 ± 9.042	−8.485	<0.001
FPG	6.035 ± 2.586	6.511 ± 3.193	−9.541	<0.001
TG	1.371 ± 0.722	1.723 ± 1.038	−9.697	<0.001
TBIL	13.181 ± 12.1	13.099 ± 5.17	−0.103	0.918
TC	5.487 ± 0.983	5.525 ± 1.08	−0.657	0.511
LAP	38.979 ± 28.265	56.306 ± 40.734	−12.302	<0.001

LAP: lipid accumulation product; WC: waist circumference; NC: neck circumference; WHR: weight-to-height ratio; WNR: weight-to-neck circumference ratio; CHD: coronary heart disease; HBP: high blood pressure; TG: triglyceride; TC: total cholesterol; FPG: fasting plasma glucose; TBIL: total bilirubin; SBP: systolic blood pressure; DBP: diastolic blood pressure.

**Table 2:** Baseline characteristics of males and females.

Variables	Male (*n* = 971)		*P*	Female (*n* = 1121)		*P* value
	NT	HT		NT	HT	
Age (years)	72.67 ± 6.622	72.83 ± 6.065	>0.05	73.19 ± 7.137	73.97 ± 6.832	<0.05
Smoker (%)	14.62%	11.53%	<0.05	1.24%	2.14%	>0.05
Drinker (%)	15.55%	13.69%	>0.05	1.96%	2.14%	>0.05
CHD (%)	4.53%	10.30%	<0.001	6.15%	11.32%	<0.001
HD (%)	5.66%	9.78%	<0.001	4.28%	13.2%	<0.001
Height (cm)	166.16 ± 6.086	166.97 ± 6.014	>0.05	154.38 ± 5.993	154.81 ± 5.715	>0.05
Body weight (kg)	63.34 ± 10.210	68.20 ± 9.824	<0.001	54.83 ± 9.324	59.31 ± 9.766	<0.001
BMI (kg/m^2^)	22.92 ± 3.448	24.43 ± 3.034	<0.001			<0.001
WC	85.656 ± 9.267	90.268 ± 8.493	<0.001	83.05 ± 10.614	87.53 ± 9.489	<0.001
NC	37.18 ± 2.793	38.45 ± 2.680	<0.001	33.71 ± 2.692	34.94 ± 2.667	<0.001
WHR	0.52 ± 0.057	0.54 ± 0.05	<0.001			<0.001
WNR	2.3 ± 0.171	2.34 ± 0.165	<0.001			<0.05
SBP	128.41 ± 15.098	139.13 ± 15.279	<0.001	130.36 ± 14.407	140.04 ± 15.514	<0.001
DBP	76.69 ± 8.109	80.47 ± 9.231	<0.001	75.73 ± 7.943	78.82 ± 8.824	<0.001
FPG	6.04 ± 1.518	6.43 ± 1.643	<0.001	6.02 ± 1.687	6.57 ± 1.895	<0.001
TG	1.29 ± 0.708	1.63 ± 1.044	<0.001	1.44 ± 0.727	1.8 ± 1.027	<0.001
TBIL	14.09 ± 5.442	14.62 ± 5.45	>0.05	12.35 ± 5.801	11.85 ± 4.571	>0.05
TC	5.23 ± 0.929	5.21 ± 1	>0.05	5.72 ± 0.970	5.78 ± 1.075	>0.05
LAP	34.24 ± 26.417	49.74 ± 27.182	<0.001	43.32 ± 29.217	61.68 ± 43.714	<0.001

NT: normotension; HT: hypertension; LAP: lipid accumulation product; WC: waist circumference; NC: neck circumference; WHR: weight-to-height ratio; WNR: weight-to-neck circumference ratio; CHD: coronary heart disease; HD: history of diabetes; TG: triglyceride; TC: total cholesterol; FPG: fasting plasma glucose; TBIL: total bilirubin; SBP: systolic blood pressure; DBP: diastolic blood pressure.

**Table 3:** Logistic regression analysis of factors associated with hypertension.

Variables	OR	95% CI	*P* value
*Overall*			
LAP	1.012	1.008–1.015	<0.001
NC	1.034	1.000–1.070	0.05
FPG	1.101	1.039–1.166	<0.01
BMI	1.062	1.025–1.099	<0.01

*Males*			
WC	1.052	1.036–1.068	<0.001
TG	1.440	1.203–1.723	<0.001

*Females*			
FPG	1.134	1.048–1.226	<0.01
LAP	1.011	1.006–1.016	<0.001
BMI	1.074	1.032–1.118	<0.001

LAP: lipid accumulation product; NC: neck circumference; FPG: fasting plasma glucose; BMI: body mass index; WC: waist circumference; TG: triglyceride.

**Table 4:** Spearman correlation analysis between LAP and related factors.

Variables	LAP	BMI	WHR	WNR	FPG	WC	NC	TG
LAP	1.000	0.615^*∗∗∗*^	0.656^*∗∗∗*^	0.475^*∗∗∗*^	0.313^*∗∗∗*^	0.724^*∗∗∗*^	0.366^*∗∗∗*^	0.869^*∗∗∗*^
NC	0.366^*∗∗∗*^	0.565^*∗∗∗*^	0.411^*∗∗∗*^	0.163^*∗∗∗*^	0.2^*∗∗∗*^	0.729^*∗∗∗*^	1.000	0.180^*∗∗∗*^
FPG	0.313^*∗∗∗*^	0.257^*∗∗∗*^	0.257^*∗∗∗*^	0.156^*∗∗∗*^	1.000	0.282^*∗∗∗*^	0.2^*∗∗∗*^	0.255^*∗∗∗*^
BMI	0.615^*∗∗∗*^	1.000	0.805^*∗∗∗*^	0.47^*∗∗∗*^	0.257^*∗∗∗*^	0.836^*∗∗∗*^	0.565^*∗∗∗*^	0.300^*∗∗∗*^
WC	0.724^*∗∗∗*^	0.836^*∗∗∗*^	0.929^*∗∗∗*^	0.703^*∗∗∗*^	0.282^*∗∗∗*^	1.000	0.729^*∗∗∗*^	0.334^*∗∗∗*^
TG	0.870^*∗∗∗*^	0.327^*∗∗∗*^	0.297^*∗∗∗*^	0.166^*∗∗∗*^	0.262^*∗∗∗*^	0.334^*∗∗∗*^	0.318^*∗∗∗*^	1.000

Notes: number means correlation coefficient; WHR: weight-to-height ratio; WNR: weight-to-neck circumference ratio; ^*∗∗∗*^*P* < 0.001.

**Table 5:** Multivariate logistic regression analysis of factors associated with hypertension.

	OR †	95% CI	OR ‡	95% CI
*Overall*				
LAP	1.017	1.014–1.021^*∗∗∗*^	1.011	1.007–1.015^*∗∗∗*^
NC	1.117	1.087–1.148^*∗∗∗*^	1.208	1.109–1.315^*∗∗∗*^
FPG	1.203	1.134–1.277^*∗∗∗*^	1.099	1.037–1.164^*∗∗*^
BMI	1.147	1.117–1.178^*∗∗∗*^	1.115	1.080–1.151^*∗∗∗*^

*Male*				
LAP	1.019	1.013–1.024^*∗∗∗*^	1.011	1.005–1.017^*∗∗*^
WC	1.060	1.044–1.076^*∗∗∗*^	1.049	1.033–1.067^*∗∗∗*^
TG	1.681	1.401–2.017^*∗∗∗*^	1.470	1.214–1.781^*∗∗∗*^
FPG	1.177	1.079–1.283^*∗∗∗*^	1.060	0.969–1.159
BMI	1.157	1.110–1.206^*∗∗∗*^	1.112	1.059–1.168^*∗∗∗*^

*Female*				
LAP	1.017	1.013–1.021^*∗∗∗*^	1.012	1.007–1.017^*∗∗∗*^
FPG	1.226	1.129–1.331^*∗∗∗*^	1.127	1.043–1.218^*∗∗*^
BMI	1.140	1.102–1.180^*∗∗∗*^	1.127	1.081–1.175^*∗∗∗*^
WC	1.046	1.033–1.059^*∗∗∗*^	1.035	1.022–1.049^*∗∗∗*^
TG	1.681	1.428–1.979^*∗∗∗*^	1.457	1.234–1.719^*∗∗∗*^

Notes: ^†^unadjusted; ^‡^adjusted for physical activity, smoker, drinker, and age (additionally, LAP is adjusted for WNR, NC, FPG, CHD, and HD; NC is adjusted for LAP WHR, WNR, FPG, and TG; FPG is adjusted for LAP, WHR, WNR, BMI, NC, and TG; BMI is adjusted for WNR, FPG, and TG; WC is adjusted for FPG and TG; TG is adjusted for BMI, WHR, WNR, FPG, WC, and NC). CHD: coronary heart disease; HD: history of diabetes; ^*∗*^*P* < 0.05; ^*∗∗*^*P* < 0.01; ^*∗∗∗*^*P* < 0.001.

**Table 6:** Predicting performance of factors in hypertension.

Variables	Cutoff value	Sensitivity (%)	Specificity (%)	Youden index	AUC (95% CI)	Model quality	*P*
*Overall*							
LAP	33.5	70.2	51.9	0.221	0.655	0.63	<0.001
NC	35.2	61.4	53.2	0.146	0.601	0.58	<0.001
FPG	5.88	53.6	65.7	0.193	0.624	0.60	<0.001
BMI	24.52	48.3	70.6	0.193	0.632	0.61	<0.001

*Male*							
LAP	36.72	57.5	65.8	0.223	0.663	0.63	<0.001
BMI	24.4	50	71.7	0.217	0.642	0.61	<0.001
FPG	5.91	50.6	66.2	0.168	0.608	0.57	<0.001
WC	89.5	55.2	66.4	0.216	0.644	0.61	<0.001
TG	1.37	51.7	68	0.197	0.626	0.59	<0.001

*Female*							
LAP	56.76	46.4	75.7	0.221	0.65	0.62	<0.001
FPG	5.88	54.9	66.4	0.213	0.637	0.61	<0.001
BMI	24.55	48.8	68.6	0.174	0.624	0.59	<0.001
WC	80.5	77.5	38.9	0.164	0.616	0.58	<0.001
TG	1.54	51.2	66.4	0.176	0.617	0.58	<0.001

AUC: area under the curve.

**Table 7:** Pairwise comparison of ROC of AUC with indexes diagnostic power.

Variables	BMI		FPG		NC	
	Chi-square and *P* value		Chi-square and *P* value		Chi-square and *P* value	
LAP	5.10	0.00239^*∗∗*^	5.09	0.0241^*∗*^	16.4	0.0001^*∗∗∗*^

^*∗*^*P* < 0.05, ^*∗∗*^*P* < 0.01, and ^*∗∗∗*^*P* < 0.001.

**Table 8:** The incremental diagnostic performance of LAP for predicting hypertension.

Variables	LAP		BMI			NC				TG		WC			FPG		
	Cox and Shell *R*^2^ (former)/Nagelkerke *R*^2^ (latter)																

LAP	0.65	0.86		0.77	0.102		0.73	0.98	0.67		0.89		0.74	0.99		0.71	0.95

## Data Availability

The data used to support the findings of this study are available from the corresponding author upon request.
